# Knowledge, attitudes and practices of sheep owners regarding abortion in Northern Tunisia

**DOI:** 10.1002/vms3.1418

**Published:** 2024-05-31

**Authors:** Afef Jeljli, Obaid Allah Ben Abid, Atef Jlassi, Ines Hammami, Mohamed Gharbi

**Affiliations:** ^1^ Departement of Sciences and Pathology of Animal Reproduction Institution of Agricultural Research and Higher Education National School of Veterinary Medicine of Sidi Thabet University of Manouba Sidi Thabet Tunisia; ^2^ Laboratory of Parasitology Institution of Agricultural Research and Higher Education National School of Veterinary Medicine of Sidi Thabet University of Manouba Sidi Thabet Tunisia; ^3^ Veterinary Practitioner Nefza Beja Tunisia

**Keywords:** abortion, attitudes, ewes, knowledge, practices, Tunisia

## Abstract

**Background:**

Abortion in ewes causes high economic losses and represents a threat for human health due to abortive zoonotic pathogens.

**Objective:**

The present study aimed to assess the knowledge, attitudes and practices (KAP) among sheep owners in the northern Tunisia regarding ewes’ abortions.

**Methods:**

Between February 2021 and May 2022, a structured questionnaire containing both close and open‐ended questions was applied to 120 sheep owners in northern Tunisia. The data collected were analysed by chi‐square test using Epi info 6 software.

**Results:**

The majority (75%) of participants reported a history of abortion in their sheep flocks. Sheep owners thought that the most frequent cause of abortion was physical factors, such as trauma, climate and stress (60% ± 5.5%; 48/80), followed by toxicity (15% ± 4%; 12/80), metabolic and nutritional conditions (12.5% ± 3.7%; 10/80), vaccination (5% ± 2.4%; 4/80) and infectious causes (7.5% ± 2.9%; 6/80) (*p *< 0.001). The majority of animal owners reported that abortions occurred mainly during autumn (39.6% ± 5%; 38/96), followed by summer (27% ± 4.5%; 26/96), winter (23% ± 4.3%; 22/96) and spring (10.4% ± 3.1%; 10/96) (*p* < 0.001). Approximately, half (45.8% ± 5%; 55/120) of interviewed farmers would not take any action if an abortion occurred. Half of the interviewed farmers (50.5% ± 5.1%; 48/95) did not apply any preventive measures when manipulating aborted ewes, and most of the sheep owners (77.3% ± 3.8%; 92/119) did not know that aborted ewes could transmit zoonotic pathogens.

**Conclusions:**

Our survey concluded that sheep owners in Northern Tunisia had poor knowledge and attitudes as well as applied limited actions concerning several health aspects related to abortion. Education programmes should be established in order to improve Tunisian sheep owners’ KAP regarding abortion.

## Graphical abstract

Our study revealed that sheep owners in Northern Tunisia lacked knowledge on several health aspects and needed an educational program to enhance their knowledge, attitudes and practices regarding sheep abortion. These findings could be used to develop and implement control programs targeting abortion in sheep in Tunisia.

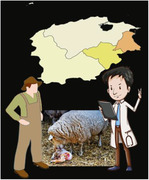

## 1 INTRODUCTION

Sheep farming plays an important role in the Tunisian economy and food security. With approximately 6.8 million sheep heads, among them 60% are females, sheep farming is one of the oldest and most widely practiced agricultural activities in Tunisia. Moreover, this activity is the main source of income for Tunisian farmers providing 45% of total Tunisian red meat production, corresponding to 120,000 MT but only 5% of Tunisian milk production (Observatoire National de l'Agriculture, 2020). As in several other African countries, Tunisian sheep are facing several health issues, such as viral diseases (foot and mouth disease, border disease and bluetongue disease) (Bouguedour & Ripani, 2016; Thabti et al., 2005), bacterial diseases (brucellosis and clostridial infections) (Barkallah et al., 2017) and parasitic diseases (toxoplasmosis, gastrointestinal parasites, lungworm nematodes and tick‐borne infections) (Akkari et al., 2011, 2012; Khamassi Khbou et al., 2021; Lahmar et al., 2012).

Abortion in ewes is a serious recurrent health issue for Tunisian sheep industry stakeholders including sheep owners, veterinary services and veterinary practitioners in two situations: (i) if it is expressing important diseases, either zoonotic (brucellosis) or highly contagious (border disease) and (ii) if the incidence of abortion is high. In the other cases, sheep owners consider that abortion is a normal event that does not need any investigation, treatment or prevention measures for the other ewes of the flock. The underestimation of this very important syndrome makes abortion a neglected sheep health issue causing high economic losses in relation to both their direct and indirect costs. Direct costs include loss of lambs, milk yield decrease and gynaecological complications leading sometimes to infertility and even to sterility (Haif et al., 2021). On the other hand, indirect costs are high but paradoxically underestimated by both sheep owners and animal health specialists. These costs are related to an increase in lambing intervals with all its consequences, including reduction in lamb production, increase in food expenses and increase of veterinary fees (Esmaeili et al., 2022). Furthermore, abortion in sheep can have great impact on public health as several sheep abortive causative agents are zoonotic such as *Brucella* spp., *Salmonella* spp., *Campylobacter* spp., *Chlamydia abortus*, *Listeria monocytogenes*, *Coxiella burnetii* and *Toxoplasma gondii*. This zoonotic capacity increases dramatically the importance of ewes’ abortion syndrome.

Sheep flock management, including good housing, respect of general hygiene measures and correct feeding and watering, can reduce sheep abortion incidence (Alemayehu et al., 2021). Nevertheless, abortion is a very complicated syndrome having a plethora of causes that could be divided into non‐infectious (nutritional, physical, toxic, chemical, iatrogenic and hormonal causes) and infectious (viral, bacterial, parasitic and fungal causes) (Haif et al., 2021).

According to a limited number of published studies, abortive agents are highly prevalent in Tunisian sheep population. They include mainly toxoplasmosis (19.7%), Q fever (17.2%) and brucellosis (16.1%) (Guesmi et al., 2023). The educational level of Tunisian farmers remains predominantly low. Indeed, Khamassi Khbou et al. (unpublished data) reported that 90% of interviewed Tunisian farmers had a primary school education level.

For these reasons, we investigated herein the knowledge, attitudes and practices (KAP) about sheep abortion through interviews targeting Tunisian sheep owners. This survey is the first step that will pave the way for adapted animal health control programme's implementation to reduce the impact of sheep abortion on both human and animal health.

## 2 MATERIALS AND METHODS

### 2.1 Study area

**FIGURE 1:**
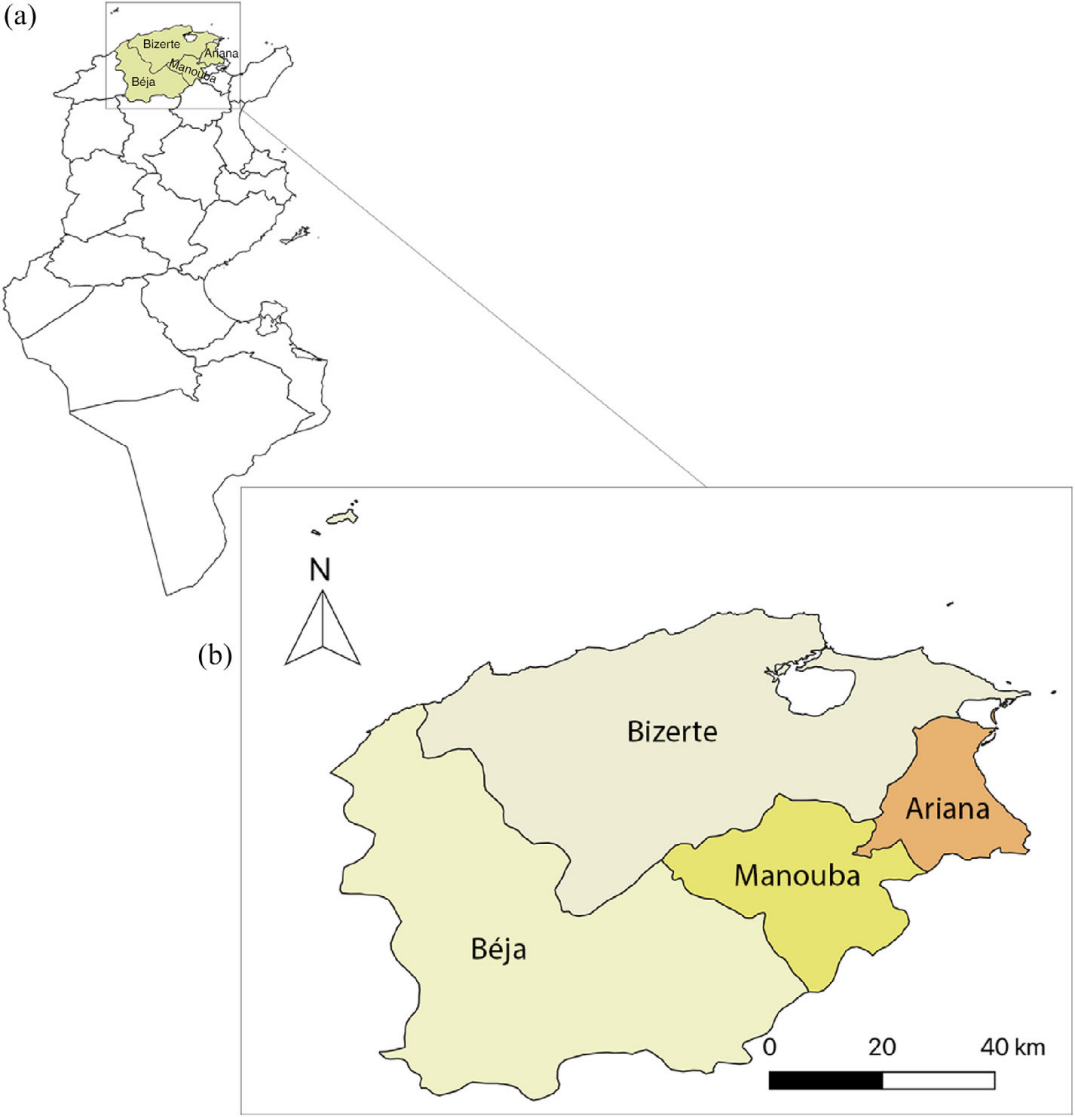
(a) Map of Tunisia (b) geographic localization of the four governorates where the present study was realized.

The present study was carried out between February 2021 and May 2022 in four governorates located in North Tunisia, namely Ariana, Manouba, Béja and Bizerte (Figure 1, Table 1).

**TABLE 1:** Characteristics of the studied governorates (http://www.onagri.nat.tn/; https://fr.climate‐data.org/).

	Governorate			
Indicator	Ariana	Manouba	Béja	Bizerte
***Area (km^2^)***	482	1137	3740	3750
***Bioclimatic zone***	Csa	Csa	Csa	Csa
***Mean altitude (m above sea level)***	23	67	223	33
***Mean annual pluviometry (mm)***	444	379	528	547
***Mean annual temperature (°C) (range)***	18.6 (8.8–31)	18.4 (7–34)	17.5 (5.6–32.8)	18.4 (9.9–29)
***Human population***	601,800	379,518	303,032	568,219
***Livestock population***				
Cattle	15,000	14,000	75,040	73,170
Sheep	34,500	98,700	343,800	219,540
Goats	2250	6740	30,750	28,790

### 2.2 Data collection

A verbal informed consent was obtained from all interviewed sheep owners. The anonymity of the participants was ensured during the whole process with the right to be forgotten for all of them. A total number of 120 sheep owners were interviewed face‐to‐face in Tunisian dialectal Arabic.

The interview consisted of both close and open‐ended questions that were pooled in four sections: (i) socio‐demographic characteristics of sheep owners: gender, age, level of education, professional activity and experience in animal breeding; (ii) information about sheep flocks; (iii) knowledge of participants about abortion: abortion history in their flocks, possible causes of abortion and associated symptoms; (iv) attitudes and practices of the animal owners regarding abortion, the management and the awareness level of zoonotic abortive pathogens. The interview was pretested on 10 sheep owners to evaluate its fluidity, duration and clarity. It was consequently improved giving a final version that required 15 min interview.

### 2.3 Analysis of data

Collected data were stored in Microsoft Excel Spreadsheet (Microsoft Corp.). Chi‐square test was performed with Epi info 6 software (Centers for Disease Control and Prevention) to compare percentages. The difference was considered statistically significant at 5% threshold (Schwartz, 1993).

## 3 RESULTS

### 3.1 Socio‐demographic characteristics of the farmers and their sheep flocks

All the interviewed sheep owners (*n *= 120) responded to the questionnaire. They owned a total number of 4385 sheep with a mean flock size of 36.8 sheep per flock (range: 4–450 sheep). The majority of the interviewed sheep owners were from Béja (49.1% ± 5.4%; 59/120), followed by Bizerte (29.1% ± 4.9%; 35/120), Ariana (12.5% ± 3.4%; 15/120) and Manouba (9.1% ± 3.1%; 11/120).

Almost all participants were males (sex ratio males: females = 19). The average age of interviewed persons was 49.6 years (range: 21–83 years). More than half (57.5% ± 5.4%; 69/120) of the respondents were exclusively sheep farmers. The majority of the responders had primary school educational level (59.5%; 25/42), followed by high school level (26.2%; 11/42), university level (9.5%; 4/42) and illiterate (4.8%; 2/42) (*p* > 0.05) (Table 2). More than half of the sheep owners refused to provide information about their educational level (65% ± 5.2%; 78/120).

**TABLE 2:** Sociodemographic characteristics of the interviewed sheep owners.

			Positive/interviewed (percentage ± SE)	
Variables	Categories	Flocks with no abortion history	Flocks with abortion history	Total
***Gender***	Male	29/120 (24.2 ± 3.9)	85/120 (70.8 ± 4.1)	114/120 (95 ± 2)
	Female	1/120 (0.83 ± 0.8)	5/120 (83.3 ± 1.8)	6/120 (5 ± 2)
***Age category (years)***	<35	5/120 (4.2 ± 1.8)	17/120 (14.2 ± 3.2)	22/120 (18.3 ± 3.5)
	35–64	18/120 (18.3 ± 3.3)	58/120 (48.3 ± 4.6)	76/120 (63.3 ± 4.4)
	≥65	8/120 (6.7 ± 2.3)	14/120 (11.7 ± 2.9)	22/120 (18.3 ± 3.5)
***Governorate***	Ariana	2/120 (1.5 ± 1.2)	13/120 (10.8 ± 2.8)	15/120 (12.5 ± 3)
	Béja	18/120 (15 ± 3)	41/120 (34.2 ± 4.3)	59/120 (49.2 ± 4.6)
	Bizerte	4/120 (3.3 ± 1.6)	31/120 (25.8 ± 4)	35/120 (29.2 ± 4.1)
	Manouba	6/120 (5 ± 2)	5/120 (41.2 ± 1.8)	11/120 (9.2 ± 2.6)
***Educational level***	Illiterate	0/42	2/42 (4.8 ± 3.3)	2/42 (4.8 ± 3.3)
	Primary school	9/42 (21.4 ± 6.3)	16/42 (38.1 ± 7.5)	25/42 (59.5 ± 7.6)
	High school	3/42 (7.1 ± 4)	8/42 (19 ± 6.1)	11/42 (26.2 ± 6.8)
	University	2/42 (4.7 ± 3.3)	2/42 (4.7 ± 3.3)	4/42 (9.5 ± 4.5)
	No response	16/120 (13.3 ± 3.1)	62/120 (51.7 ± 4.6)	78/120 (65 ± 4.4)
***Occupation***	Public sector	3/120 (2.5 ± 1.4)	5/120 (4.2 ± 1.8)	8/120 (6.7 ± 2.3)
	Private sector	6/120 (5 ± 2)	32/120 (26.7 ± 4)	38/120 (31.7 ± 4.2)
	Sheep owner	18/120 (15 ± 3.3)	51/120 (42.5 ± 4.5)	69/120 (57.5 ± 4.5)
	Farmer	2/120 (1.7 ± 1.2)	3/120 (2.5 ± 1.4)	5/120 (4.2 ± 1.8)
***Experience in sheep breeding (years)***	1–10	9/120 (7.5 ± 2.4)	24/120 (20 ± 3.7)	33/120 (27.5 ± 4.1)
	11–20	6/120 (5 ± 2)	6/120 (5 ± 2)	12/120 (10 ± 2.7)
	21–30	3/120 (2.5 ± 1.4)	7/120 (5.8 ± 2.1)	10/120 (8.3 ± 2.5)
	31–40	2/120 (1.7 ± 1.2)	14/120 (11.7)	16/120 (13.3 ± 3.1)
	41–51	3/120 (2.5 ± 1.4)	16/120 (13.3 ± 3.3)	19/120 (15.8 ± 3.3)
	≥51	7/120 (5.8 ± 2.1)	23/120 (19.2 ± 3.6)	30/120 (25 ± 4)
***Type of breeding system***	Intensive	29/120 (24.2 ± 3.9)	88/120 (73.3 ± 4)	117/120 (97.5 ± 1.4)
	Extensive	1/120 (0.8 ± 0.8)	2/120 (1.7 ± 1.2)	3/120 (2.5 ± 1.4)
***Herd size***	≤20	23/119 (19.3 ± 3.6)	40/119 (33.6 ± 4.3)	63/119 (52.9 ± 4.6)
	21–50	7/119 (5.9 ± 2.2)	35/119 (29.4 ± 4.2)	42/119 (37.8 ± 4.4)
	>50	0/119	14/119 (11.8 ± 3)	14/119 (11.8 ± 3)
	No response	0/120	1/120 (0.8 ± 0.8)	1/120 (0.8 ± 0.8)

### 3.2 Knowledge of sheep owners about abortion

The majority (75% ± 4%; 90/120) of interviewed farmers reported a history of abortion in their sheep flocks. Sheep owners thought that the most frequent cause of abortion was physical factors, such as trauma, climate and stress (60% ± 5.5%; 48/80), followed by toxicity (15% ± 4%; 12/80), metabolic and nutritional conditions (12.5% ± 3.7%; 10/80), vaccination (5% ± 2.4%; 4/80) and infectious causes (7.5% ± 2.9%; 6/80) (*p* < 0.001).

According to sheep farmers, the majority of abortions occurred during autumn (39.6% ± 5%; 38/96), followed by summer (27% ± 4.5%; 26/96), winter (23% ± 4.3%; 22/96) and spring (10.4% ± 3.1%; 10/96) (*p* < 0.001).

The majority (64.4% ± 5%; 58/90) of sheep owners did not answer the question about the clinical signs associated to abortion (*p *< 0.001). Twenty participants (62.5% ± 8.6%) reported that the expulsion of mummified brown fetus was the most common sign observed followed by mastitis and metritis (15.6% ± 7.9%; 9/32), respiratory symptoms (18.7% ± 6.9%; 6/32), neurological symptoms (1.1% ± 3.1%; 1/32) and miscellaneous symptoms (respiratory, nervous, metritis and mastitis) (43.7% ± 8.8%; 14/32) (Table 3).

**TABLE 3:** Sheep owners’ knowledge about ewes’ abortion.

Question	Farms with abortion history	Farms without abortion history	Total
***Causes of abortion sheep in general***			
Physical (trauma, climate and stress)	41/120 (34.2 ± 4.3)	16/120 (13.3 ± 3.1)	57/120 (47.5 ± 4.6)
Toxic	23/120 (19.2 ± 3.6)	9/120 (7.5 ± 2.4)	32/120 (26.7 ± 4)
Metabolic and nutritional	12/120 (10 ± 2.7)	3/120 (2.5 ± 1.4)	15/120 (12.5 ± 3)
Infectious	9/120 (7.5 ± 2.4)	1/120 (0.8 ± 0.8)	10/120 (8.3 ± 2.5)
Vaccination	3/120 (2.5 ± 1.4)	0/120	3/120 (2.5 ± 1.4)
Don't know	3/120 (2.5 ± 1.4)	0/120	3/120 (2.5 ± 1.4)
***How to identify the cause of abortion?***			
Laboratory diagnosis	3/10 (30 ± 14.5)	0/10	3/10 (30 ± 14.5)
Through field veterinarian	1/10 (10 ± 9.5)	1/10 (10 ± 9.5)	2/10 (20 ± 12.6)
Analysing animals’ food	5/10 (50 ± 15.8)	0/10	5/10 (50 ± 15.8)
Don't know	81/120 (67.5 ± 4.3)	29/120 (24.2 ± 3.9)	110/120 (91.7 ± 2.5)
***Season of most frequent abortion cases***			
Summer	23/96 (23.9 ± 4.4)	2/96 (2.1 ± 1.5)	26/96 (27.1 ± 4.5)
Autumn	28/96 (29.2 ± 4.6)	10/96 (10.4 ± 3.1)	38/96 (39.6 ± 5)
Winter	15//96 (15.6 ± 3.7)	7/96 (7.3 ± 2.7)	22/96 (22.9 ± 4.3)
Spring	7/96 (73.9 ± 2.7)	3/96 (3.1 ± 1.2)	10/96 (10.4 ± 3.1)

### 3.3 Attitudes and practices of sheep owners towards abortion

Approximately half (45.8% ± 5%; 55/120) of interviewed farmers did not take any action when an abortion occurred, 15% ± 3.3% (18/120) provided supplementary food to aborted ewes, 13.3% ± 3.1% (16/120) isolated systematically the aborted female, 10% ± 2.7% (12/120) called a veterinarian and 10% ± 2.7% (12/120) treated their aborted ewes with ethnopharmaceutical drugs (they use mainly fenugreek, olive oil and olive tree leaves). Auto‐medication (they gave systemic and local antibiotics) was performed by 9.1% ± 2.6% (11/120) and 6.6% ± 2.3% (8/120) culled aborted ewes. Half of the interviewed farmers (50.5% ± 5.1%; 48/95) did not observe any preventive measures when manipulating aborted ewes, 26.3% ± 4.5% (25/95) wore gloves, 7.3% ± 2.7% (7/95) handled aborted fetuses using plastic bags; 14.7% ± 3.6% (14/95) washed their hands after manipulating aborted fetuses. Finally, only 1% ± 1% (1/95) of the farmers did not handle aborted fetuses (*p* < 0.001).

### 3.4 Knowledge of sheep owners about abortion preventive measures

Only 35% ± 4.4% (42/120) of interviewed sheep owners knew that several vaccines against abortive pathogens were available, but 7.5% ± 2.4% (9/120) of participants did not answer this question (*p* < 0.001).

### 3.5 Knowledge of sheep owners about abortion and associated zoonotic risk

Most of sheep owners (77.3% ± 3.8%; 92/119) did not know that aborted ewes could transmit zoonotic infections. Moreover, the majority of respondents (83.2% ± 3.4%; 99/119) did not know that abortion in sheep constituted a zoonotic risk for women. Only 20 respondents (16.7% ± 3.4%) were aware of the risk of transmission of pathogens to women when handling an aborted ewe. Almost all (19/20) of sheep owners thought that handling an aborted animal represented a zoonotic risk without knowing its nature. One respondent indicated that the simple contact of pregnant women with an aborted ewe or its fetus represents a risk for these women. Educational level, experience in sheep breeding, locality, herd size and knowledge about abortion causes showed statistically significant associations with the presence of abortion cases (*p* < 0.05).

## 4 DISCUSSION

To our knowledge, the present study is the first in Tunisian sheep flocks exploring the knowledge and practices of farmers about sheep abortion. All interviewed 120 Tunisian sheep owners responded to the survey, indicating that sheep abortion constituted a major concern for all of them. The majority (75%; 90/120) of interviewed sheep owners reported abortion cases in their flocks. Similar rates were reported by other authors in Algeria (between 75.33% and 90%) (Khaled & Bouyoucef, 2013; Kardjadj et al., 2016) and in Turkey (85%) (Yilmaz et al., 2002). The rate we obtained here was higher than that reported by Aldomy and Abu Zeid (2007) in a study that was carried out in Jordan (53.5%).

Interviewed sheep owners thought that sheep abortion was attributable to several causes and that the most frequent was physical cause, such as trauma, climatic conditions and stress (52.5%), followed by toxicity (15%), metabolic and nutritional issues (12.5%) and infectious causes (7.5%).

In Tunisia, there is a gap of knowledge about sheep abortive infectious causative agents that could be attributed to a lack of laboratory diagnostic tools. Borel et al. (2014) recommended an investigative approach for each abortion case in ruminants consisting of (i) registration of the case history, (ii) collecting blood from the aborting female for serology, (iii) performing a macroscopic examination of the fetus and placental membranes, (iv) collecting samples from fetuses and placenta for microbiology, histopathology and molecular analyses, (v) performing routine bacteriology (vi) and histopathological examination, (vii) using immunohistochemistry or in situ hybridization to investigate the presence of pathogens and (viii) immediate reporting of abortions. However, this protocol requires optimal conditions for its application, and in many cases, it is hampered by several factors, including lack of money, uncertainty of etiological diagnosis, transportation of aborted fetuses and placentas and availability of equipped laboratory (Brom et al., 2021). In Tunisia, abortion is not a notifiable syndrome in all animal species and studies conducted on Tunisian sheep abortion are limited to some regions (Khamassi Khbou et al., 2009).

A serological study carried out by Elandalousi et al. (2015) in northern Tunisia showed that sheep were infected mainly by *C. burnetii* (56.84%), followed by *Chlamydia* spp. and *Brucella* spp. (10.5%). A recent serological study carried out by Guesmi et al. (2023) in seven Tunisian governorates revealed that 19.7%, 17.2% and 16.1% of the tested sera were positive for *T. gondii*, *C. burnetii* and *Brucella* spp., respectively.

Most of the interviewed sheep owners (30% ± 14.5%) did not know that laboratory diagnosis allows identification of the abortion causative agent. This could be explained by a poor involvement of veterinarians in the management of sheep abortion as this etiological diagnosis requires a close and continuous collaboration among animal owners, veterinarians, medical doctors and laboratories (Holler, 2012).

Interviewed sheep owners thought that abortions occurred mainly in autumn. Similarly, Yilmaz et al. (2002) reported that in Turkey, abortion occurred mainly between November and February. Alemayehu et al. (2021) reported that the abortion incidence increased at the beginning of the short cold dry season (June to August) (Alemayehu et al., 2021). This incidence pattern could be due to the insufficient feed resources during the dry period in semi‐arid areas especially for extensively managed sheep, a common management system in Tunisia (Alemayehu et al., 2021; Rekiki et al., 2005).

In addition, during summer and autumn, heat stress could contribute to the increase of abortion incidence (Silanikove, 2000; Zhang et al., 2020). Entrican et al. (2009) indicated that heat stress and inadequate diet increase the infection risk by abortive pathogens. Exposing pregnant ewes to heat stress in summer during mid‐ and late‐gestation dramatically reduces the total embryo cell number and placentome size. In addition, lamb production is negatively correlated to heat stress (Marai et al., 2007).

Only 10% of sheep owners asked for veterinary assistance after an abortion case. A higher percentage was reported in Turkey (38%) (Yilmaz et al., 2002) and in Algeria where more than half of the interviewed animal owners called their veterinarians (55%) (Khaled & Bouyoucef, 2013). This low percentage in Tunisia could be explained by high veterinary fees and a lack of knowledge about the role played by veterinarians in managing abortion episodes as reported by Khaled and Bouyoucef, 2013.

A total number of 16 (13.3%) animal owners systematically isolated their aborted ewes. Higher percentages were reported in Algeria (Khaled & Bouyoucef, 2013) (27%) and in Egypt, Holt et al. (2011) reported roughly the same percentage in cattle (10.3%) but it was twice higher in small ruminants (23.8%). The low observance of this important management recommendation indicates a lack of knowledge about transmission mechanisms of abortive pathogens (Menzies, 2011).

Due to an important lack of knowledge about zoonotic risks associated with some sheep abortive pathogens, more than half of sheep owners did not apply preventive measures when getting in contact with infectious materials. Indeed, only 26.3% of interviewed sheep owners wore gloves when manipulating aborted fetuses. A similar proportion was reported by Khaled and Bouyoucef, 2013 in Algeria (31%). In Egypt, all the interviewed farmers did not wear gloves (Holt et al., 2011).

Most sheep owners (77.3%; 92/119) were not aware of the zoonotic risk related to abortion in sheep. In Algeria, Khaled and Bouyoucef (2013) reported that almost half (49%) of farmers were aware of zoonotic risk during sheep abortion. In Egypt, Holt et al. (2011) reported that a high percentage of sheep owners were aware of these diseases, especially brucellosis (82.3%).

This lack of knowledge might have important negative impact on public health in view of the high number of abortive zoonotic pathogens in sheep that could be transmitted through tissue manipulation of aborted animals and the foetal tissues. This may emphasise the importance of veterinarians in raising awareness about abortion zoonotic diseases. For this reason, farmers should be continuously educated and advised to respect basic biosafety rules when getting any abortion case in their sheep flocks.

Only 35% (42/120) of interviewed farmers knew that there are several vaccines against abortive pathogens. In Tunisia, small ruminants are vaccinated, free of charge, against brucellosis, foot and mouth disease and bluetongue disease. These vaccinations are performed yearly as national campaigns by private veterinarians that are paid by the Tunisian Ministry of Agriculture. The vaccination against sheep abortion is an essential part of a good health management system and a key requirement in intensive systems (Esmaeili et al., 2022). This lack of knowledge might be due to a huge gap of knowledge of these diseases by Tunisian animal health professionals. For this reason, a better collaboration between laboratories, field veterinarian's animal health decision makers and sheep owners is needed to elaborate and implement adapted sheep health management programmes.

## 5 CONCLUSION

The present study revealed several gaps of knowledge about abortion among Tunisian sheep owners. In addition, they do not have correct attitudes and practices during abortion in ewes. In order to improve both human and animal health related to sheep infectious abortive pathogens, there is an urgent need for the implementation of specific educational programmes targeting and improvement of KAP of sheep owners regarding abortion.

## AUTHOR CONTRIBUTIONS

*Conceptualization; data curation; formal analysis; writing – original draft; writing – review and editing*: Afef Jeljli. *Data curation; formal analysis; investigation*: Obaid Allah Ben Abid. *Investigation*: Atef Jlassi. *Formal analysis*: Ines Hammami. *Conceptualization; supervision; writing – review and editing*: Mohamed Gharbi.

## CONFLICT OF INTEREST STATEMENT

The authors declare no conflicts of interest.

### ETHICS STATEMENT

This study was performed by certified veterinarians that respected the ethical recommendations of the Tunisian state.

### CONSENT TO PARTICIPATE

The authors obtain verbal informed consent from all sheep owners prior to conducting the interviews.

### PEER REVIEW

The peer review history for this article is available at https://www.webofscience.com/api/gateway/wos/peer-review/10.1002/vms3.1418.

## CONSENT FOR PUBLICATION

All authors have read the manuscript and consented to its publication.

## Data Availability

The data that support the findings of this study are available from the corresponding author upon reasonable request.
